# Lifeworld perspectives of people with dementia: a meta-aggregation of qualitative studies

**DOI:** 10.1186/s12877-022-03660-w

**Published:** 2022-12-15

**Authors:** Siren Eriksen, Knut Engedal, Ellen Karine Grov

**Affiliations:** 1 The Norwegian National Centre for Ageing and Health, Vestfold Hospital Trust, Tønsberg, Norway; 2 Lovisenberg Diaconal University College, Oslo, Norway; 3 Department of Geriatric Medicine, Oslo University Hospital, Oslo, Norway; 4 Department of Nursing and Health Promotion, Oslo Metropolitan University, Oslo, Norway

**Keywords:** Dementia, Lifeworld, Interview, Experience, Space, Relations, Body, Time, Van Manen

## Abstract

**Objectives:**

This meta-aggregation aims to interpret and synthesize present knowledge on the lifeworld perspectives of people with dementia and develop a model for guidance in clinical practice.

**Method:**

The data consist of four meta-syntheses describing different lifeworld perspectives in accordance with van Manen’s existentials: lived relations, lived space, lived time and lived body. The meta-aggregation summarizes a range of views expressed by people with dementia in qualitative, interview-based studies, with the aim of generating a reliable model based on the studies’ findings.

**Results:**

In total, 88 studies among 1,191 persons with dementia were included. Sixteen areas of focus were found, representing four perspectives: (a) *lived relations*, consisting of connectedness, independence, equality and competence; (b) *lived space*, consisting of belonging, meaningfulness, safety and security, and autonomy; (c) *lived time*, consisting of being rooted in the past, being in the present, viewing the future and being in process; and (d) *lived body*, consisting of being functional, trustworthy, adaptable and presentable. A model shaped as a tree trunk captures the lifeworld perspectives of people with dementia.

**Conclusion:**

Sixteen areas were revealed from this meta-aggregation and form the basis of a model. This model may be used as a guide for health care personnel to ensure the overall lifeworld-perspectives of people with dementia in care for the target group and conduct lifeworld-preserving care with a person-centred approach.

## Introduction

In a report about dementia prevention, intervention and care for the Lancet commission in 2020, Livingston and co-authors stated that promoting well-being of people with dementia is one of the main goals of dementia care [1]. Further, they state that people with dementia have complex problems and symptoms in many domains and well-being is therefore multifaceted. We would add that a more precise term would be to state they have complex needs. From a natural sciences perspective, dementia is a progressive syndrome, characterized by specific symptoms affecting the person’s functioning and health status as well as their ability to manage daily life activities—thus leading to a medical diagnosis (ICD-11). From a human sciences perspective, dementia is an illness as experienced by the person living with the syndrome. Illness refers to how the person with dementia and their relatives or wider social network perceive, live with and respond to their functional decline, symptoms and disabilities. From a social sciences perspective, dementia is a sickness understood as a syndrome in a generic sense across a population, in relation to economic, political, cultural and institutional forces [2, 3]. Well-being is an entirely personal experience and interventions should therefore be individualized, and the individual treated as a whole person. This ‘wholeness’ requires all three perspectives, natural sciences, human sciences and social sciences [4]. This paper is based in human science, emphasizing the lifeworld perspective of people with dementia.

Human activity is always an oriented and directed performance. For a person living with a disease or syndrome it cannot be experienced isolated from the context: the person will sense, feel, dream and judge the experience in relation to his/her world [5]. This world, as it reveals itself to our consciousness, is what Husserl [6] has termed the *lifeworld*. This is a different world than the objective, outer world. Although human experiences of that lifeworld are deeply personal, they also constitute essential meanings or *essential structures of a phenomenon*. Here, structures refer to the shared world of being a human and can be revealed through in-depth descriptions of individual experiences [7]. In other words, when investigating the experiences of several persons with a particular disease or syndrome (for example, dementia that is caused by various neurological diseases), one might find similar or different essential structures but all of them refer to the shared (familiar) experience of being human. A phenomenological perspective in research emphasizes human experience—i.e., the lifeworld as it reveals itself through communicating about one’s experiences—, as the basis of human science, leading us to essential structures of human lives [8].

Based on a phenomenological and pedagogical perspective and in order to describe and constitute the complexity of the lifeworld, the philosopher Max van Manen [5] identified four fundamental existential themes in which the lifeworld may be seen: (a) lived relation to others, (b) lived space, (c) lived time, and (d) lived body. Through our *lived relation* to other persons (‘lived others’)*,* we share an ‘interpersonal space’ with those around us; moreover, when we meet other persons, we may develop a conversational relationship, which allows us to transcend ourselves. The experience of *lived space* is described as the ‘felt space’; our experience of space is often pre-verbal, in contrast to mathematical space or the length, depth or height dimensions of space. The experience of *lived time* covers the experience of perceived time as opposed to clock time or objective time; lived time describes our temporal way of being in the world, where the three dimensions of past, present and future constitute the horizons of a temporal landscape. The experience of the *lived body* positions the body by describing people as living in the world with the body and living and being in relation with others through the body. van Manen terms these four essential structures *existentials, as we interpret as perspectives of the lifeworld*. They may be reflected upon separately; however, it is only together, as a whole, that they constitute the lifeworld. van Manen asserts that if a person’s experience of one existential change, their experience of the others will also change—as will their experience of their entire lifeworld [5].

The lived experience of dementia has been studied with different approaches, at different stages of the syndrome and in different contexts. Qualitative, interview-based studies have shown that the experience of losing the ability and the right to decide for oneself, lacking control over the body and daily life, and feeling different than others and not ‘normal’ in one’s own and others’ eyes is common when living with dementia [9–12].

To gain knowledge about the essential structures of living with dementia, a group of researchers led by the first author of this article (SE) conducted a systematic literature search for interview-based studies of people with dementia. Due to the number of studies and the complexity of the field, we decided to analyse the data in relation to van Manen’s four existentials. This resulted in one article describing each of the four perspectives. In the present meta-aggregation, we take it further and interpret and synthesize the experience of lived relations, lived space, lived time and lived body to describe and aggregate the essential structures of the lifeworld of people with dementia.

## Methods

We selected articles in accordance with the lifeworld framework developed by van Manen. We searched for articles covering studies using qualitative interviews. If studies included spouses or health care personnel in addition to people with dementia, the person with dementia’s voice had to be emphasized. Intervention studies were excluded.

**Fig. 1:**
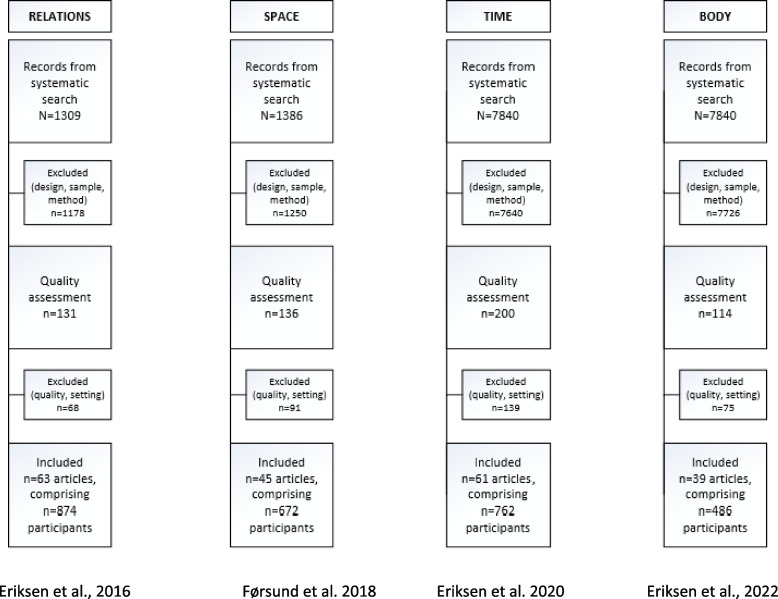
Flowchart of the process of identification, screening, eligibility and inclusion of articles and participants to this lifeworld study

The data consist of four meta-syntheses describing the different lifeworld perspectives in accordance with van Manen’s existentials: specifically, lived relations [9], lived space [10], lived time [11] and lived body [12]. Each of the four published articles contain a specific description of the literature search and a presentation of the inclusion and exclusion process addressed in PRISMA descriptions with corresponding flowcharts [13]. Additionally, quality assessment was performed using the Critical Appraisal Skills Program’s (CASP) criteria for qualitative studies to score and document the included studies [14]. A flowchart is attached to show the inclusion and exclusion process for the four meta-syntheses (see Fig. 1). For further descriptions of the method, please see the four meta-syntheses forming the basis of this meta-aggregation [9–12].

To aggregate the findings from the meta-syntheses into a synopsis, the analysis followed the principles of interpretive synthesis in three steps: (a) overall description of the categories described in each meta-synthesis; (b) integration of the categories; and (c) synthesis with the new interpretations of the phenomenon (i.e., the lifeworld perspectives of people with dementia) derived from synthesizing all the reports [15]. This meta-aggregation summarizes a range of views and enables generalizable statements appropriate for health care workers by means of reliable presentations of the findings from the included studies. We performed a verbatim extraction to categorize and create new perspectives and theoretical issues; we did not, however, re-interpret the four studies [16]. In this way, we strived for a transparent presentation [17]. The outcome is an explanatory illustration (model) based on the participants’ voice drawing on van Manen’s four lifeworld perspectives.

In total, 88 studies with 1,191 people with dementia were included, 77 of which included people living at home, in their own home or ordinary housing (hereafter ‘own home’), and 11 included people living in long-term care, a nursing home, dementia care unit, assisted living or residential care home (hereafter, ‘long-term care’). all of the four lifeworld perspectives were not present in all 88 articles. Thus, different numbers of participants are included in the different meta synthesis.

## Results

The main results present the different lifeworld perspectives in accordance with van Manen’s existentials. The main results have been published earlier in four separate and different meta-syntheses: specifically, lived relations [9], lived space [10], lived time [11] and lived body [12].

### Lived relations

Living with dementia changes one’s life, leading to new social roles and a changed social status. The process of adapting to these changes is essential for a good life. A person with dementia may attempt to compensate for a lack of ability to normalize the situation and ‘cover up’ the dementia. Moreover, new connections (e.g., peers) and more longstanding relationships (e.g., family members) play significant roles in maintaining important aspects of a meaningful life. Our analysis revealed four categories describing important relational aspects:

- *Disconnected*: Many people with dementia experienced that the relationships they had with others were changing due to the dementia. Feeling disconnected represented a distance from social relations and familiar activities. The experience of withdrawal, discontinuity, isolation, restricted freedom and homesickness (for those living in long-term care) was sometimes present. This distance may be understood as either wanting to be disconnected from others, or as others being disconnected from them.
- *Dependent:* Being dependent was felt to be a consequence of functional impairment in activities of daily living and reduced cognitive and social function for the person with dementia.
- *Burden:* Being a burden was expressed as being sensitive to the consequences for their family, friends and caregivers.
- *Treated:* Being treated in paternalistic ways captures how family, friends and health care personnel often behaved towards the person. Being treated in this way denoted care without choices or the ability to influence the care being provided [9].

### Lived space

Our analysis shows that living in one’s own home and living in long-term care posed diverse challenges for the experience of lived space. As dementia progresses, it seems like lived space gradually becomes smaller. Nevertheless, people with dementia continue to experience the spatial dimensions of their lifeworld through their lived space. The data reveal four main categories that describe the experience of lived space: not only the physical and social environment, but also space in an existential way:

A. *Belonging:*
  - Own home: People with dementia considered living at home and in their own home to be very important for their experience of belonging.
  - Long-term care: Belonging was described as the experience of being familiar with the setting and feeling like they are in the right place. Home was perceived as the key to living a good and meaningful life. Moving into long-term care appeared to cause disorientation in some cases and challenged people’s overall sense of belonging. Admittance to long-term care was described as the ‘beginning of the end’, or as the start of a new life that signalled an overall ‘winding down’.
B. *Meaningfulness:*
  - Own home: Home was described as a centre for meaning and a place for retreat, solitude, rejuvenation, socialization, connectedness and affiliation, and a centre for meaningful activities of daily living.
  - Long-term care: Meaningfulness was related to one’s ability to be occupied with interesting and relevant activities. Some experienced long-term care as an important arena for social activities, others longed for privacy or were bored.
C. *Safety and security:*
  - Own home: Home was described as a venue where one could avoid stress and do things at their own pace, a place where one was safe and secure, an arena for coping, comfort and continuity in relation to traditions and social life.
  - Long-term care: Safety in long-term care was related to a sense of being safe and comforted by others (health care personnel). Long-term care was understood as a place for hospitality and rest.
D. *Autonomy:*
  - Own home: Many perceived that living in their own home was the locus of autonomy, control, choice and the freedom to act. They highlighted the importance of being able to take care of themselves.
  - Long-term care: Lack of autonomy was related to the process of moving and to everyday life in long-term care. Life in long-term care was monotonous with lost abilities and loss of freedom, few individual opportunities, lack of privacy and uncertainty [10].

### Lived time

Our study found that people with dementia are engaged with the different dimensions of time—the past, the present and the future—and that they experience changes in self related to all three dimensions. Thus, the experience of lived time is an active and important one, in terms of enabling people to process and manage the dementia journey. Four categories emerged from the material:

- *Rooted in the Past:* People with dementia often leaned towards the past to make sense of the present: i.e., ‘I am the same as before’. Rooting themselves in the past by looking back and reliving events seemed to make it easier for them to accept their current life situation and compensate for what they may have lost.
- *Focusing on the present:* By focusing on being in the here and now, people with dementia could simultaneously leave the past behind and avoid thinking about the future. Living with a progressive syndrome enhanced the need to live in the moment, as one’s future was no longer certain.
- *Thinking about the future:* Some people with dementia spent time thinking about the days to come. They perceived the future as a time of uncertainty and inevitable demise.
- *Changes in experiences of self over time:* People with dementia noticed changes in their experiences of self over time. The phrase ‘used to’ features a great deal in the data, as participants described how their character has altered over time due to the dementia [11].

### Lived body

The results pointedly describe the heterogeneity of the impact of dementia and ageing on physical function. The inability to comprehend one’s bodily changes can cause feelings of fear and loss of control. But some describe the body as a resource and an access to the world and to participation. The lived body experience has relational aspects and others’ behaviour may affect one’s experience of the body; one can feel approved or denounced. Four categories emerged from the material:

- *My body works:* People with dementia described themselves as physically healthy and experienced that their functioning body allowed them to stay connected to others and society.
- *My body betrays me:* On the other hand, they often expressed the experience of loss of physical function and a fear of becoming helpless. The experience of being different than before and feeling a lack of confidence was prominent.
- *Understanding and adapting to my body’s changes:* Many found it difficult to understand the dementia syndrome, and this was experienced as distressing. Some managed to actively cope with the diagnosis by making an effort to take control and keep going.
- *My body in relation to others:* In this perspective, people with dementia described the experience of their bodies as being perceived and seen by others, how they compare their body to other people’s bodies and how they feel objectified because of the dementia [12].

### The synopsis: areas of focus

**Fig. 2:**
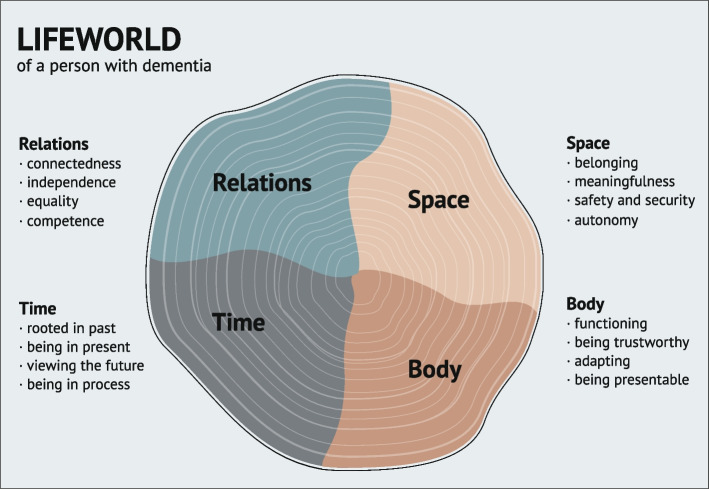
The synopsis: areas of focus

To aggregate the underlying categories of lived relations, space, time and body as comprising essential lifeworld perspectives of people with dementia, we have extracted the findings into 16 areas of focus, 4 for each of the existentials (as shown in Fig. 2):

- RELATIONS: connectedness, independence, equality, competence
- SPACE: belonging, meaningfulness, safety and security, autonomy
- TIME: rooted in the past, being in the present, viewing the future, being in process
- BODY: functioning, being trustworthy, adapting, being presentable

## Discussion

In dementia care, a primary goal should be to improve wellbeing. As people with dementia have complex needs and symptoms in a wide range of domains, interventions should be individualised, and the person considered as a whole [1]. The experience of one’s lifeworld depends on the experience of the four existentials and the underlying areas of focus. van Manen [5] argues that these existentials are universal and shared by all humans, which appears true for the people with dementia in the present study. As noted earlier, he also states that the existentials are intertwined: if a person’s experience of one existential change, their experience of the others—and thus their entire lifeworld—will change as well.

In the interviews presented in the papers and used in the categorization, people with dementia often point at what they find challenging or what they are lacking. They are less concerned with describing what they need, which raises the question: Is it always possible to know what one needs? In response, we argue that the participants’ challenges, wishes and requirements as reported in each article can be understood as their expressed fundamental and overall needs, and designated as *areas of focus for the lifeworld of people with dementia*. For instance, the experience of being disconnected reveals the importance of being connected and describes the challenging space between connection and disconnection. And the experience of a betraying body reveals the importance of having a trustworthy body, emphasizing the nuances between trustworthiness and betrayal. These contrasts make us aware of the importance of having experienced one perspective to gain awareness or knowledge about a diametral opposite. Correspondingly, in a study by Staats et al. [18], the authors present the participants’ way of describing the concept of dignity by explaining their experiences as a loss of dignity. These *areas of focus* are described in Fig. 2; in the figure, the outer edge of the tree trunk represents the frame of the person’s lifeworld, and the yearlings symbolize their lived life and their history in context. These areas are of major importance to supporting lifeworld-preserving care for people with dementia.

The interview-based studies included in this aggregation show that many people with dementia have insight into their own situation and that they are often able to express this, even at moderate and severe stages of the syndrome. For vulnerable people like those with dementia, some experiences and needs will be experienced as threatened —for example, safety and security. However, first and foremost our study indicates that people with dementia think and react as most people do and that their perspectives appear quite similar to those of people without a cognitive syndrome. Indeed, many of the needs incorporated in the model are universal.

According to the World Alzheimer Report from 2019, an international study of attitudes towards people with dementia, the majority of people believe that those with dementia are impulsive and unpredictable (63.6%) and dangerous (16.8%)—and these attitudes inform the ways in which people interact with those with dementia. The same study shows that 85% of people with dementia do not think that their opinion is taken seriously [19]. The dementia syndrome also often takes over as the main descriptor of the person: the person with dementia becomes the syndrome [19]. Moreover, social stigma of people with dementia is a global problem leading to negative consequences, such as rejection, discrimination and exclusion [20]. Kelly and Innes [21] emphasize a lack of awareness about human rights and citizenship in dementia care. People with dementia may be exposed to neglect, lack of privacy and dignity, insufficient attention paid to confidentiality, inappropriate medication and use of restraint or discrimination on the grounds of age, disability or race [21]. These violations of human rights ‘affront human dignity and identity’ [22].

Working to counter these issues, person-centred care is well-established as an important element of dementia care [4], promoting individuals’ dignity, identity and human rights, and opposing stigma. The essence of person-centered care is to acknowledge personhood, meeting every person with respect and as a valuable person regardless of age and cognitive abilities. In dementia care, an important tenet of the person-centered care approach is to take into consideration the viewpoint of the person with dementia [4]. Van Manen has constructed a theoretical framework with four existentials significant for human lifeworld. In our four published articles on these four existentials, we have shown that the construct is highly relevant for clinical practice and issues concerning people with dementia. Thus, the lifeworld perspective should form the basis for person-centred, non-pharmacological interventions for people with dementia, to help them live a good life, continue to be him/herself and experience being in a familiar lifeworld. Our work shows that people with dementia are able to express and describe their needs and wishes in different stages of the dementia syndrome. The 16 areas of focus presented in our model could guide person-centred care and thus help health care personnel to individualise care and maintain the persons lifeworld as a whole. By bearing the areas of focus in mind, health care personnel could for instance help the person with dementia to express unmet needs and wishes within the different areas.

We have chosen to use the theoretical framework developed by van Manen to develop a comprehensive outline of the lifeworld perspectives of people with dementia, as shown in Fig. 2. This model could guide person-centred care by emphasizing the aspects of importance in the four lifeworld perspectives. Through the model, the voices of people with dementia are made clear.

## Conclusion

This study is based on the theoretical framework of van Manen which describes four lifeworld perspectives: lived relations, lived space, lived time and lived body. The method used in this paper is inspired by the approach used in meta-aggregation. The model developed from the aggregation of the categories present in this study illustrates the lifeworld existentials as expressed by people with dementia. This model could serve as a guide for health care personnel to secure lifeworld-preserving care with a person-centred approach for people with dementia. Further, the study indicates that people with dementia think and react as most people do and that their lifeworld perspectives do not significantly differ from of people without a cognitive syndrome.

## Data Availability

All data generated or analysed during this study are included in this published article.
